# Artificial intelligence in bone age assessment: accuracy and efficiency of a novel fully automated algorithm compared to the Greulich-Pyle method

**DOI:** 10.1186/s41747-019-0139-9

**Published:** 2020-01-28

**Authors:** Christian Booz, Ibrahim Yel, Julian L. Wichmann, Sabine Boettger, Ahmed Al Kamali, Moritz H. Albrecht, Simon S. Martin, Lukas Lenga, Nicole A. Huizinga, Tommaso D’Angelo, Marco Cavallaro, Thomas J. Vogl, Boris Bodelle

**Affiliations:** 1 Division of Experimental Imaging, Department of Diagnostic and Interventional Radiology, University Hospital Frankfurt, Theodor-Stern-Kai 7, 60590 Frankfurt am Main, Germany; 2 Department of Diagnostic and Interventional Radiology, University Hospital Frankfurt, Frankfurt am Main, Germany; 3 Interdisciplinary Center for Neuroscience, Goethe-University of Frankfurt, Frankfurt am Main, Germany; 4 Department of Biomedical Sciences and Morphological and Functional Imaging, University of Messina, Messina, Italy

**Keywords:** Age determination by skeleton, Algorithms, Artificial intelligence, Image processing (computer-assisted), Retrospective studies

## Abstract

**Background:**

Bone age (BA) assessment performed by artificial intelligence (AI) is of growing interest due to improved accuracy, precision and time efficiency in daily routine. The aim of this study was to investigate the accuracy and efficiency of a novel AI software version for automated BA assessment in comparison to the Greulich-Pyle method.

**Methods:**

Radiographs of 514 patients were analysed in this retrospective study. Total BA was assessed independently by three blinded radiologists applying the GP method and by the AI software. Overall and gender-specific BA assessment results, as well as reading times of both approaches, were compared, while the reference BA was defined by two blinded experienced paediatric radiologists in consensus by application of the Greulich-Pyle method.

**Results:**

Mean absolute deviation (MAD) and root mean square deviation (RSMD) were significantly lower between AI-derived BA and reference BA (MAD 0.34 years, RSMD 0.38 years) than between reader-calculated BA and reference BA (MAD 0.79 years, RSMD 0.89 years; *p* < 0.001). The correlation between AI-derived BA and reference BA (*r* = 0.99) was significantly higher than between reader-calculated BA and reference BA (*r* = 0.90; *p* < 0.001). No statistical difference was found in reader agreement and correlation analyses regarding gender (*p =* 0.241). Mean reading times were reduced by 87% using the AI system.

**Conclusions:**

A novel AI software enabled highly accurate automated BA assessment. It may improve efficiency in clinical routine by reducing reading times without compromising the accuracy compared with the Greulich-Pyle method.

## Key points

- A novel artificial intelligence software enabled highly accurate bone age (BA) assessment.
- AI allowed for reading time reduction without compromising the accuracy.
- Efficiency of BA assessment in daily routine may be improved by artificial intelligence.

## Background

Bone age (BA) assessment is common in paediatric radiology, being a clinically relevant parameter for estimating biological and skeletal maturity [1, 2]. High accuracy of BA assessment is required for an exact diagnosis and optimal therapy of several paediatric disorders. Frequent indications for BA assessment include microsomia, macrosomia, *pubertas tarda*, and *pubertas praecox* as well as therapy with growth hormone [1–3]. Furthermore, it is commonly applied for forensic investigations if birth records are unavailable.

Currently, there are several methods for BA assessment such as the Greulich-Pyle and the Tanner-Whitehouse methods [1–3], the former being mostly used. It is based on an atlas consisting of 57 reference radiographs (31 for males, 26 for females). Radiographs of the left hand and wrist are compared with the reference radiographs in the atlas. Widespread availability and cost-effectiveness are main advantages of this approach. However, numerous disadvantages prevail such as time-demand and subjective image evaluation, which makes this method highly dependent on the reader’s expertise [1, 4]. Furthermore, this approach is not population-specific and contemporaneous since it is based on reference radiographs from a Caucasian population [1, 3]. In addition, there are long time intervals concerning the reference images, which prevent an exact statement about BA in certain cases.

In order to objectify BA assessment and to make it more efficient in daily routine, numerous artificial intelligence (AI) systems have been developed [5–10]. Thodberg et al. [10–14] initially investigated a fully automated BA assessment software tool. This software takes a more differentiated approach, analysing radiographs of the left hand and wrist and assessing BA values of 13 bones including ulna, radius, and 11 short bones in fingers 1, 3, and 5 [10]. Bone shape, density scores, and texture features are important parameters for this algorithm to identify and differentiate osseous structures. The radiograph analysis is divided into three subsequent layers. Initially, the software defines bones of interest by application of active appearance models. In a second step, bone Ag for each defined bone is determined and validated. In the final step, the software transforms the calculated BA values into Greulich-Pyle or Tanner-Whitehouse BA values.

Previous studies have demonstrated promising results from initial versions of this software in Caucasian children showing an overall accuracy in terms of mean absolute deviation (MAD) *versus* the reference standard of 0.71 years [4, 5, 15–17]. Studies investigating initial software versions of this AI system in Japanese children demonstrated an overall accuracy of 0.72 years [17, 18]. After the development of a novel software version (version 2.1), Zhang et al. [6] demonstrated excellent efficiency (98.8%) and substantially improved overall accuracy (MAD 0.64 years for boys) in Chinese children. According to these results, we hypothesised that the novel software version also enables substantially improved accuracy and efficiency in clinical routine for automated BA assessment in German children. Thus, the purpose of our multireader study was to assess the accuracy and efficiency, including reading time measurements, of a novel AI BA assessment software version by comparing it to Greulich-Pyle-based assessment in a German population and to investigate whether the accuracy is dependent on gender.

## Methods

### Patient selection and study design

This retrospective study was approved by the institutional review board, and informed consent was waived. We included 533 German patients who had undergone clinically indicated radiographs of the left hand and wrist between January 2015 and January 2016. We excluded patients older than 17 years (*n* = 8), patients younger than 3 years (*n* = 9) and patients with known malignancy of the left hand (*n* = 2). Thus, 514 patients were ultimately evaluated. Frequent indications for the radiographs in this study were microsomia (*n* = 218), macrosomia (*n* = 112), and *pubertas praecox* (*n* = 52). Table 1 summarises detailed patient characteristics.

**Table 1:** Detailed characterisation of the patient cohort in this study (*n* = 514)

Characteristics	Value
Age, years, mean ± standard deviation (min, max)	10.20 ± 4.85 (3, 17)
Male, *n* (%)	252 (49.0)
Female, *n* (%)	262 (51.0)
Known indications for the examinations	
*Pubertas praecox*, *n* (%)	52 (10.1)
Acute lymphoblastic leukaemia, *n* (%)	19 (3.7)
Therapy with growth hormone, *n* (%)	14 (2.7)
Macrosomia, *n* (%)	112 (21.8)
Microsomia, *n* (%)	218 (42.4)
Other diseases, *n* (%)	99 (19.3)

### Data acquisition

All radiographs of the left hand and wrist were performed using default settings according to the manufacturer in this study. A Multix Top ACSS x-ray system with a Polydoros SX 65/80 generator and an Optitop 150/40/80 HC x-ray tube (Siemens Healthineers, Erlangen, Germany) was applied, and all radiographs were performed by application of a copper filter (thickness of 0.1 mm) in posterior-anterior acquisition.

### Bone age assessment

**Fig. 1:**
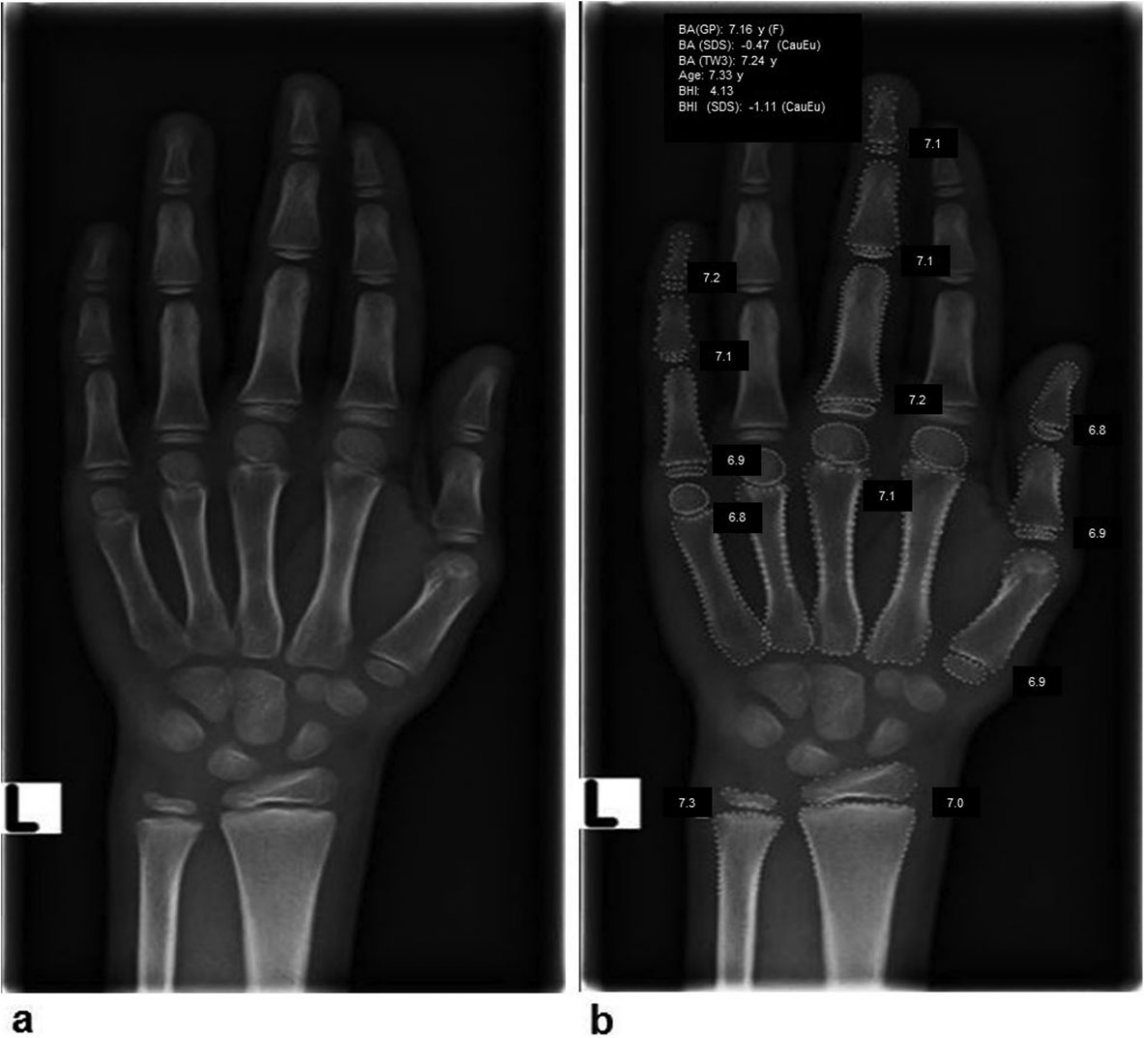
Radiograph of the left hand and wrist before (**a**) and after (**b**) automated bone age (BA) assessment by the software BoneXpert version 2.1. Bone borders of the analysed bones are automatically marked by the software. The software first calculates the intrinsic overall BA and the BA of each bone. Afterwards, these intrinsic values are transformed into Greulich-Pyle and Tanner-Whitehouse values (version TW3). The values are displayed in the annotated DICOM (Digital Imaging and Communications in Medicine) image as transformed Greulich-Pyle bone age values (BA [GP]) and Tanner-Whitehouse values (BA [TW3]) with respect to sex (F, female). Furthermore, the bone health index (BHI) is also shown with corresponding values. In addition, the standard deviation score (SDS, also known as *z*-score) is displayed concerning the corresponding population (CauEu, Caucasian European). *BA (GP)* Greulich-Pyle bone age, *BA (SDS)* Bone age standard-deviation score, *BA (TW3)* Tanner-Whitehouse bone age, *BHI* Bone health index, *BHI (SDS)* Bone health index standard-deviation score, *CauEu* Caucasian European, *F* Female

**Fig. 2:**
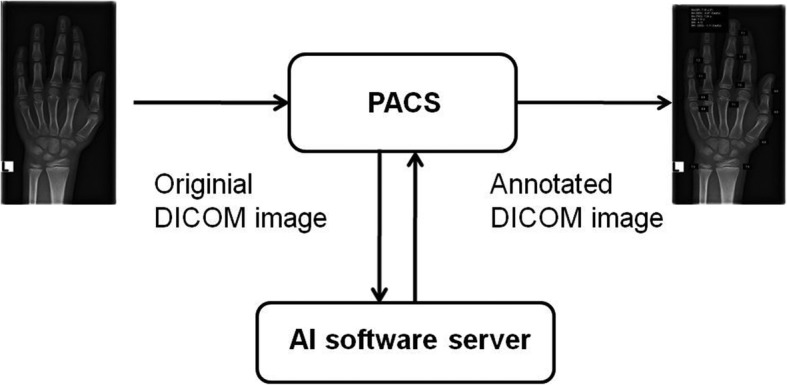
Workflow of the evaluated software BoneXpert version 2.1. After sending the radiograph in DICOM (Digital Imaging and Communications in Medicine) format to the AI (artificial intelligence) software server, the software calculates the bone age of 13 bones (radius, ulna, and 11 short bones in fingers 1, 3, and 5) by application of active appearance models, which have learned the regular shape and density distribution of each analysed bone. After this analysis, the annotated DICOM image is sent back automatically to the PACS (Picture Archiving and Communication System). *AI* Artificial intelligence, *DICOM* Digital Imaging and Communications in Medicine, *PACS* Picture Archiving and Communication System

For automated BA assessment, dedicated AI software (BoneXpert version 2.1; Visiana, Holte, Denmark) was applied, which enables BA assessment based on radiographs of the left hand and wrist. As previously described, the BA of 13 bones (radius, ulna, and 11 short bones in fingers 1, 3, and 5) is assessed by this software (Fig. 1). After the Digital Imaging and Communications in Medicine (DICOM) image is sent to the AI software server, the software first reconstructs and validates bone margins by application of active appearance models, which have learned the regular shape and density distribution of each analysed bone (layer A). Then, the intrinsic BA of each identified bone is assessed, and the total intrinsic BA is calculated by averaging the intrinsic values (layer B). In a last step, the AI system transforms intrinsic values into Greulich-Pyle or Tanner-Whitehouse values (layer C). The AI-based assessment itself is not based on the Greulich-Pyle or Tanner-Whitehouse method. After completing the analysis, the annotated DICOM image is sent to the Picture Archiving and Communication System (PACS) (Fig. 2). BA values deviating more than 2.4 years from the average of all assessed bones are deemed unacceptable by the software. If fewer than eight bones are identified and analysed by the software, the radiograph is rejected. In addition, images suffering from poor image quality or showing abnormal bone morphology are rejected automatically.

To define the reference BA, two experienced paediatric radiologists (T.J.V., head of department with 31 years of experience in paediatric imaging, and S.B., senior attending with 25 years of experience in paediatric imaging) analysed the radiographs in consensus using the Greulich-Pyle method. In addition, all radiographs were analysed independently by three radiologists with varying levels of experience in paediatric imaging (B.B., board-certified radiologist with 10 years of experience; M.H.A., radiology resident with 5 years of experience; I.Y., radiology resident with 4 years of experience) applying the Greulich-Pyle method. The three radiologists separately assessed total BA values. All readers were blinded to clinical information, chronological age, and the results of AI-based assessment. In addition, reading times of each reader as well as of the AI software were noted.

### Statistical analysis

Statistical analysis was performed by applying dedicated software (SPSS, Version 21; IBM, Armonk, New York; and MedCalc for Windows, Version 13; MedCalc Software, Mariakerke, Belgium). The normality of data was assessed by application of the Kolmogorov-Smirnov test. All variables are expressed as mean ± standard deviation. A *p* value less than 0.05 was considered as showing significant difference.

The correlation analysis in this study was performed by computing Pearson product-moment correlation and Bland-Altman plot as well as further regression analyses. The concordance correlation coefficient *ρc* and the bias correction factor *Cβ* were calculated for assessing the precision and accuracy. The concordance correlation coefficient *ρc* was interpreted as follows: *ρc* < 0.90, poor agreement; *ρc* = 0.90–0.95, moderate agreement; *ρc* = 0.95–0.99, substantial agreement; and *ρc* > 0.99, almost perfect agreement [19]. In order to determine the agreement between the investigated BA assessment methods, the root-mean-square deviation (RMSD) and mean absolute deviation (MAD) were calculated. Inter-reader agreement among the three different radiologists assessing BA by application of the Greulich-Pyle method was assessed using Fleiss’ *κ*. The *κ* value was interpreted as follows: *κ* < 0.20, poor agreement; *κ* = 0.21–0.40, fair agreement; *κ* = 0.41–0.60, moderate agreement; *κ* = 0.61–0.80, good agreement; and *κ* = 0.81–1.0, excellent agreement.

## Results

A total of 514 radiographs of German children performed between January 2015 and January 2016 were included and analysed, consisting of 262 girls (mean chronological age, 10.10 ± 3.65 years; range, 3–17 years) and 252 boys (mean chronological age, 10.31 ± 3.93 years; range, 3–17 years). Mean overall chronological age was 10.20 ± 4.85 years (range, 3–17 years). The Kolmogorov-Smirnov test demonstrated normality of the age class distribution of the patient population in this study with *D* = 0.0512 (*p* = 0.043) (Table 2).

**Table 2:** Summary of main results

Characteristics	Overall	Boys	Girls
Number of patients	514	252	262
Mean chronological age ± SD	10.20 ± 4.85	10.31 ± 3.93	10.10 ± 3.65
Mean reference BA ± SD	9.91 ± 2.85	9.95 ± 2.89	9.87 ± 2.87
Mean AI-derived BA ± SD	9.98 ± 2.94	9.99 ± 2.98	9.96 ± 2.91
Accuracy of AI-derived BA	MAD 0.34 RMSD 0.38	MAD 0.34 RMSD 0.40	MAD 0.33 RMSD 0.36
Mean GP-based BA ± SD	10.18 ± 3.94	10.21 ± 3.88	10.15 ± 3.97
Accuracy of GP-based BA	MAD 0.79 RMSD 0.89	MAD 0.79 RMSD 0.90	MAD 0.80 RMSD 0.88

Apart from number of patients, all values are given in years

*AI* Artificial intelligence, *BA* Bone age, *GP* Greulich-Pyle, *MAD* Mean absolute deviation, *RMSD* Root mean square deviation, *SD* Standard deviation

### Total BA assessment

**Fig. 3:**
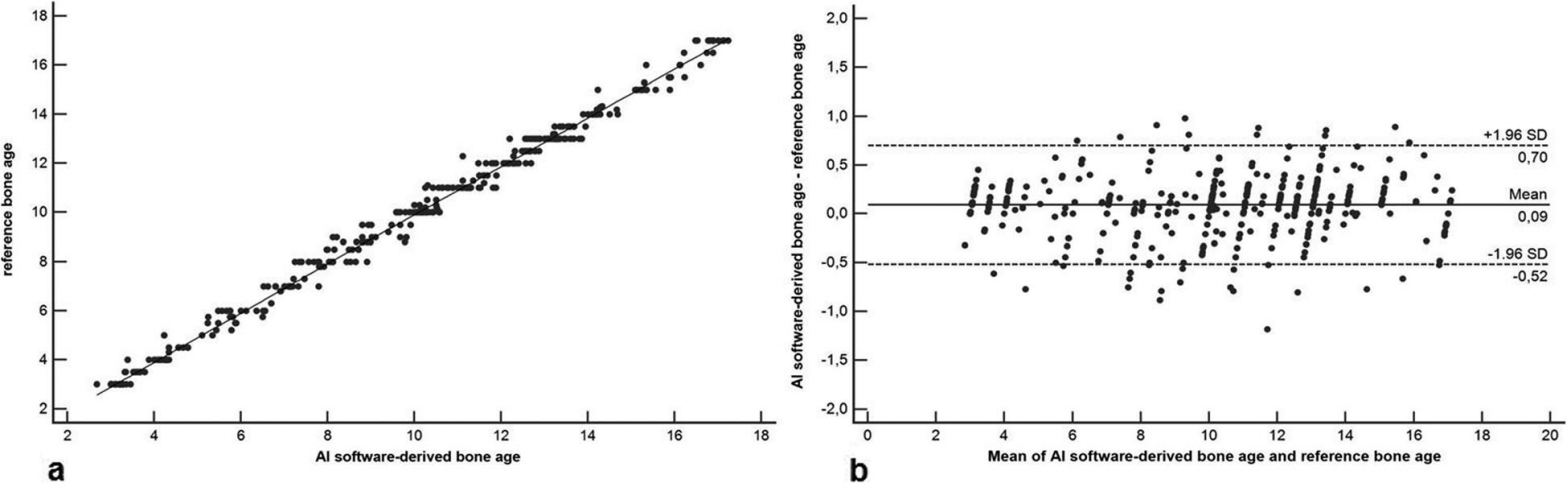
Correlation and agreement analysis of BoneXpert version 2.1-derived BA values and the reference values. **a** Pearson product-moment correlation shows excellent correlation with *r* = 0.988, 95% confidence interval (CI) 0.971–0.998, and *r*^*2*^ = 0.976, 95% CI 0.951–0.992. **b** Bland-Altman plot demonstrates excellent agreement between BA values of both approaches. Values are given in years. *AI* Artificial intelligence, *BA* Bone age

**Fig. 4:**
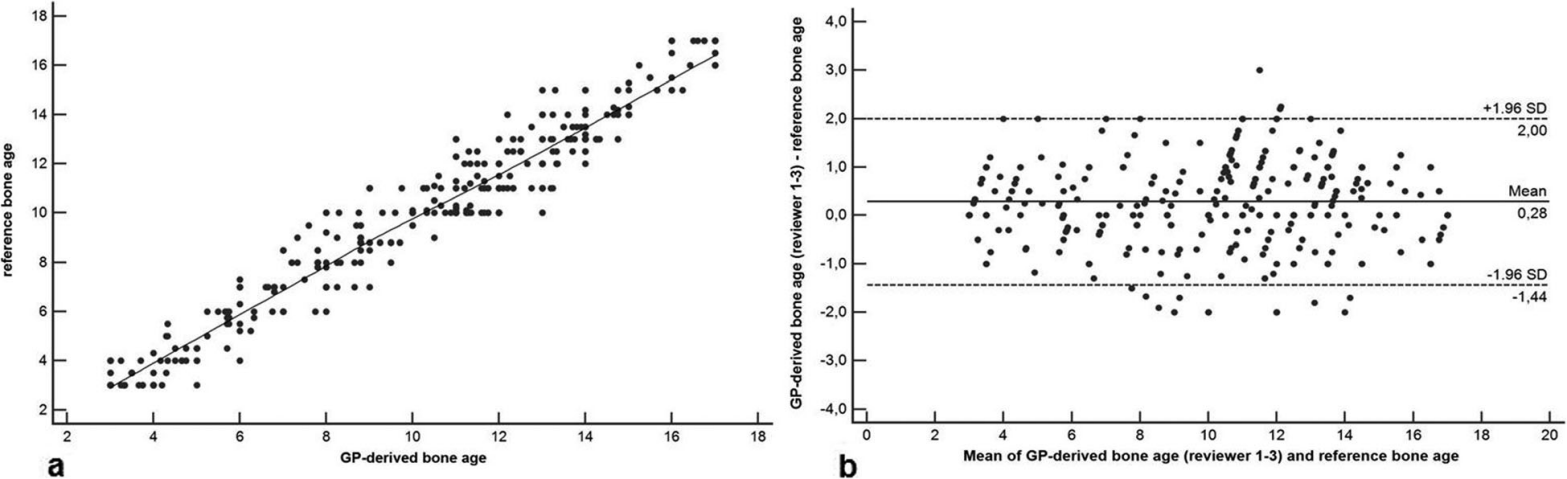
Correlation and agreement analysis of bone age values assessed by the three radiologists applying the Greulich-Pyle method and the reference values. **a** Pearson product-moment correlation shows significantly lower correlation with *r* = 0.901, 95% confidence interval (CI) 0.872–0.934, and *r*^*2*^ = 0.812, 95% CI 0.762–0.854, compared to the correlation between values based on the software and the reference values (*p* < 0.001). **b** Bland-Altman plot also demonstrates lower agreement between values assessed by the three radiologists applying the Greulich-Pyle method and the reference values compared to the agreement between values based on the software and the reference values (*p* < 0.001). Values are given in years. *GP* Greulich-Pyle

**Fig. 5:**
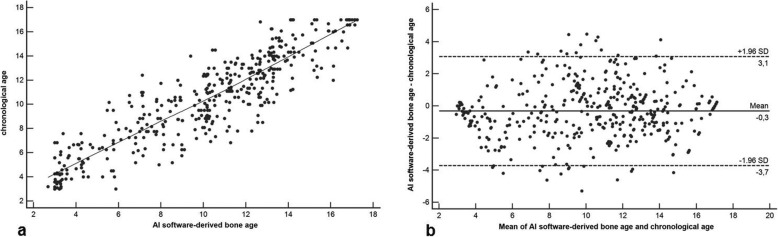
Correlation and agreement analysis of BoneXpert version 2.1-derived BA values and chronological age values. **a** Pearson product-moment correlation demonstrates excellent correlation with *r* = 0.912, 95% confidence interval (CI) 0.856–0.965, and *r*^*2*^ = 0.832, 95% CI 0.801–0.862. **b** Bland-Altman plot shows excellent agreement between software-derived BA values and chronological age values. Values are given in years. *AI* Artificial intelligence, *BA* Bone age

**Fig. 6:**
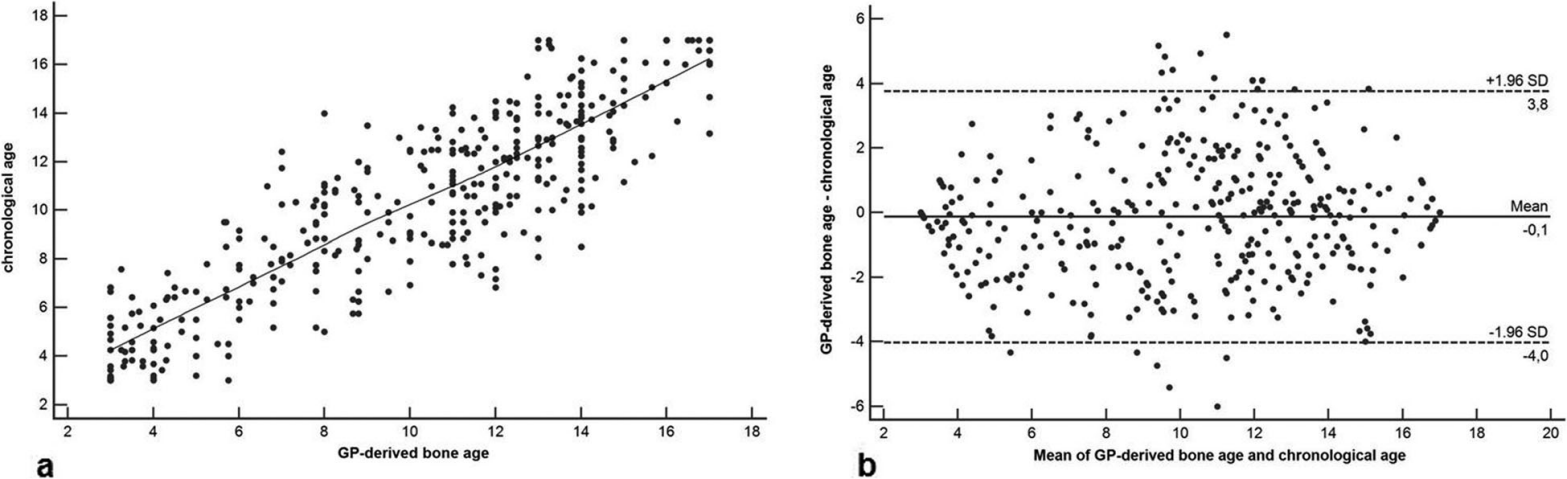
Correlation and agreement analysis of BA values assessed by the three radiologists applying the Greulich-Pyle method and chronological age values. **a** Pearson product-moment correlation demonstrates significantly lower correlation with *r* = 0.842, 95% confidence interval (CI) 0.803–0.902, and *r*^*2*^ = 0.760, 95% CI 0.725–0.804, compared to the correlation between values based on the software and chronological age values (*p* < 0.001). **b** Bland-Altman plot also shows lower agreement between BA values assessed by the three radiologists applying the Greulich-Pyle method and chronological age values compared to the agreement between values based on the software and chronological age values (*p* < 0.001). Values are given in years. *BA* Bone age, *GP* Greulich-Pyle

The reference standard calculated a mean reference BA of 9.91 ± 2.85 years (girls, 9.87 ± 2.87 years; boys, 9.95 ± 2.89 years). Mean AI-derived BA was 9.98 ± 2.94 years (girls, 9.96 ± 2.91 years; boys, 9.99 ± 2.98 years). BA assessment based on the Greulich-Pyle method assessed by the three radiologists yielded a mean BA of 10.18 ± 3.94 years (girls, 10.15 ± 3.97 years; boys, 10.21 ± 3.88 years) on average. The inter-reader agreement was excellent with a *κ* value of 0.88. There was a significantly higher correlation between AI-based BA and reference values (*r* = 0.988, 95% confidence interval [CI] 0.971–0.998; *r*^*2*^ = 0.976, 95% CI 0.951–0.992) compared to values determined by the three radiologists and reference values (*r* = 0.901, 95% CI 0.872–0.934; *r*^*2*^ = 0.812, 95% CI 0.762–0.854; comparison *p* < 0.001) (Figs. 3 and 4). In addition, there was a significantly higher correlation between AI-based BA and chronological age (*r* = 0.912, 95% 0.856–0.965; *r*^*2*^ = 0.832, 95% CI 0.801–0.862) compared to BA values determined by the three radiologists and chronological age values (*r* = 0.842, 95% CI 0.803–0.902; *r*^*2*^ = 0.760, 95% CI 0.725–0.804; comparison *p* < 0.001) (Figs. 5 and 6). Regarding gender, the statistical analysis demonstrated no significant difference concerning the correlation analysis (*p* = 0.241?). In addition, the concordance correlation coefficient showed higher agreement between AI-based BA and reference values (*ρc* = 0.983, 95% CI 0.974–0.995, substantial agreement) compared to values determined by the three radiologists and reference values (*ρc* = 0.898, 95% CI 0.862–0.937, poor agreement; comparison *p* < 0.001). The bias correction factors *Cβ* were 0.991 (AI *versus* reference 95% CI 0.982–0.999) and 0.976 (Greulich-Pyle *versus* reference 95% CI 0.967–0.988; comparison *p* < 0.001). MAD was 0.34 years (AI *versus* reference 95% CI 0.15–0.54; girls, 0.33 years; boys, 0.34 years) and 0.79 years (Greulich-Pyle *versus* reference 95% CI 0.61–96; girls, 0.80 years; boys, 0.79 years). Compared with chronological age, MAD of AI BA was 0.61 years (95% CI 0.35–0.87; girls, 0.58 years; boys, 0.76 years), while MAD of Greulich-Pyle BA was 0.99 years (95% CI 0.63–1.26; girls, 0.91 years; boys, 1.11 years). RMSD was 0.38 years (AI *versus* reference BA 95% CI 0.19–0.54; girls, 0.36 years; boys, 0.40 years) and 0.89 years (Greulich-Pyle *versus* reference 95% CI 0.59–1.23; girls, 0.88 years; boys, 0.90 years), implicating significant higher agreement between AI-based and reference BA compared to BA assessed by the three radiologists using the Greulich-Pyle method and reference values. Compared with chronological age, RMSD of AI BA was 0.56 years (95% CI 0.26–0.79; girls, 0.53 years; boys, 0.59 years), while RMSD of Greulich-Pyle BA was 1.12 years (95% CI 0.92–1.34; girls, 1.06 years; boys, 1.20 years).

### Efficiency of AI-based bone age assessment

In this study, the investigated AI software was able to analyse all radiographs (*n* = 514) without any rejection. The analysis of evaluation times of both BA assessment approaches showed a significant difference between AI- and Greulich-Pyle-based bone age assessment (*p* < 0.001). Mean evaluation times were 21 s (AI-derived assessment; range, 16–27 s) and 165 s (Greulich-Pyle-based assessment; range, 123–214 s), resulting in a mean reading time reduction of 86.9% by application of the AI software compared to the GP method. In addition, AI-based assessment enabled a mean reading time reduction of 88.5% compared with the reference standard (mean reading time of both experienced paediatric radiologists using the Greulich-Pyle methods = 182 s, range 163–222 s).

## Discussion

This retrospective study evaluated the accuracy and efficiency of a novel AI software version developed for automated BA assessment in German children in comparison to the Greulich-Pyle method. The results demonstrated that the novel AI software version permits highly accurate BA assessment by automatically analysing radiographs of the left hand and wrist compared to the Greulich-Pyle method. In this context, there was very high correlation and agreement between both assessment methods regardless of gender. Interestingly, the results of AI-based assessment yielded significantly higher accuracy compared to Greulich-Pyle-based assessment performed by three independent radiologists, while the consensus results of two very experienced paediatric radiologists using the Greulich-Pyle method served as the reference standard in this study. Furthermore, our results showed that BA assessment based on the evaluated AI software substantially reduces reading time compared to the Greulich-Pyle method, potentially improving workflow efficiency in daily clinical routine.

The RMSD of total BA values based on both methods was significantly smaller in this study (overall, 0.38 years; girls, 0.36 years; boys, 0.40 years) compared to studies correlating initial versions of the investigated AI software with human ratings (overall, 0.71 years [18]; girls, 0.76 years; boys, 0.65 years [5]). These results may indicate that technical advances concerning the AI software by developing version 2.1 of BoneXpert have improved the accuracy of this approach compared to initial software versions. Nevertheless, these findings have to be re-evaluated by multicentre studies with larger patient cohorts. In this study, the software was able to automatically analyse all radiographs without any rejections. This result, implicating a high level of efficiency, was similar to that obtained by studies investigating the novel AI software version in other patient populations, reinforcing the reliability and efficiency of this version in clinical routine [6]. Furthermore, our results indicate that AI-based BA assessment, using the novel software version, markedly lowers reading time compared to the Greulich-Pyle method, potentially improving time efficiency in clinical routine without compromising the assessment accuracy. Stern et al. [20] investigated a novel AI-based BA assessment approach based on left hand magnetic resonance imaging. They demonstrated that the MAD between chronological and estimated BA was 0.85 years, which represents a greater difference as compared to our study (MAD 0.61 years). Furthermore, magnetic resonance imaging examinations performed for BA assessment are time-consuming and difficult to perform without motion artefacts especially in young children, potentially resulting in less applicability in daily routine compared to radiography-based methods.

The Greulich-Pyle method is an established, widely available and cost-effective approach for BA assessment. However, its disadvantages merit careful consideration: subjective image evaluation, time demand and high dependence on radiologist’s expertise. In addition, the Greulich-Pyle method is non-population-specific and contemporaneous given that the atlas is based on radiographs from a Caucasian population [1, 3]. In this context, numerous studies have shown improved accuracy of BA assessment by implementation of temporally and geographically specific standards [21–25]. Therefore, a contemporaneous and objective approach, which represents the investigated novel AI software version in this study, may substantially increase the accuracy of BA assessment compared to conventional methods. In addition, the long-time intervals between reference images of the Greulich-Pyle method are overcome by AI-based assessment.

There are several limitations of this study. Due to the retrospective design, in which radiographs performed at only one institution were evaluated, data of only 514 patients were analysed. In order to re-evaluate the results and conclusions of our small single-centre study, a multicentre study with a larger patient cohort is necessary. Radiographs of the left hand and wrist of patients under 3 years and over 17 years were excluded according to the manufacturer’s guidelines. The accuracy of the AI-based approach for patients outside this range remains unclear. Furthermore, clinical indications for imaging in this study (microsomia, 42.5%) may have caused a selection bias. Finally, the range of expertise and experience of the radiologists (10, 5, and 4 years) who assessed BA using the Greulich-Pyle method may have influenced the human rating and agreement analysis.

In conclusion, this study demonstrated that a novel AI software version, BoneXpert 2.1, enables highly accurate and efficient objective BA assessment, regardless of gender, in a German patient cohort compared to the Greulich-Pyle method. In relation to earlier studies, which correlated initial versions of the AI software with human BA ratings, the accuracy of this software version has significantly improved. Furthermore, this AI software substantially reduces reading times compared to the Greulich-Pyle method, potentially improving time efficiency in clinical routine without compromising the accuracy of BA assessment.

## Data Availability

The datasets used and/or analysed during the current study are available from the corresponding author on reasonable request.

## Abbreviations

AI: Artificial intelligence
BA: Bone age
CI: Confidence interval
DICOM: Digital Imaging and Communications in Medicine
MAD: Mean absolute deviation
PACS: Picture Archiving and Communication System
RMSD: Root mean-square deviation
